# Efficacy of *Dipterocarpus alatus* oil combination with *Rhinacanthus nasutus* leaf and *Garcinia mangostana* pericarps against canine demodicosis

**DOI:** 10.14202/vetworld.2021.2919-2928

**Published:** 2021-11-16

**Authors:** Atchara Artchayasawat, Parichart Boueroy, Thidarut Boonmars, Benjamabhorn Pumhirunroj, Pranee Sriraj, Ratchadawan Aukkanimart, Sirintip Boonjaraspinyo, Opal Pitaksakulrat, Panaratana Ratanasuwan, Apiporn Suwannatrai, Chatanun Eamudomkarn, Porntip Laummaunwai, Wu Zhiliang

**Affiliations:** 1 Department of Parasitology, Faculty of Medicine, Khon Kaen University, Khon Kaen 40002, Thailand; 2 Cholangiocarcinoma Research Institute, Khon Kaen University, Khon Kaen 40002, Thailand; 3 Department of Community Health, Faculty of Public Health, Kasetsart University Chalermphrakiat Sakon Nakhon Province Campus, Sakon Nakhon 47000, Thailand; 4 Program in Animal Science, Faculty of Agricultural Technology, Sakon Nakhon Rajabhat University, Sakon Nakhon 47000, Thailand; 5 Department of Traditional Medicine, Faculty of Natural Resources, Rajamangala University of Technology ISAN Sakon Nakhon Campus, Sakon Nakhon 47160, Thailand; 6 Department of Community Medicine, Faculty of Medicine, Khon Kaen University, Khon Kaen 40002, Thailand; 7 Department of Anesthesiology, Faculty of Medicine, Khon Kaen University, Khon Kaen 40002, Thailand; 8 Department of Parasitology, Gifu University School of medicine, Gifu 501-1194, Japan

**Keywords:** demodicosis, *Dipterocarpus alatus*, mangosteen pericarp, treatment, white crane flower

## Abstract

**Background and Aim:**

Canine demodicosis is a skin disease that is a major global health problem in dogs. Ivermectin is a drug of choice for treatment, but it may cause toxicity in dogs carrying multidrug resistance mutation-1 gene mutations. Hence, alternative herbal medicines are used instead of the drug, such as *Dipterocarpus alatus* oil (YN oil), *Rhinacanthus nasutus* leaf (WC), and *Garcinia mangostana* pericarps (MG) extracts. This study aimed to determine the efficacy of *D. alatus* oil, *R. nasutus* leaf, and *G. mangostana* pericarp extracts on canine demodicosis *in vivo*.

**Materials and Methods:**

Twenty-five mixed-breed dogs with localized demodicosis were examined. Dogs were diagnosed with demodicosis through deep skin scraping and screened with the inclusion criteria. Five dogs of each group were treated in five treatment groups (ivermectin, YN oil, YN oil+WC, YN oil+MG, and YN oil+WC+MG) for 1 month. The individual dogs were clinically evaluated, and the dermatological lesions were monitored daily for 60 days.

**Results:**

Dermatological lesion improvement was predominantly observed in the group of dogs treated with YN oil+WC. This was evidenced by the disappearance of the hyperpigmentation and lichenification on day 28 post-treatment and alopecia on day 56 post-treatment. Moreover, no allergic or clinical signs were observed during treatment.

**Conclusion:**

YN oil+WC is an alternative herbal medicine that could be used for the treatment of localized canine demodicosis.

## Introduction

Canine demodicosis is a parasitic skin disease in dogs that occurs worldwide, including in Thailand. It is caused by the proliferation of *Demodex canis* in the hair follicles and sebaceous glands [1]. Nevertheless, *D. canis* can also cause dermatological lesions in humans [2]. *Demodex* spp. are detected in both humans and dogs in skin scrapings and hair samples, infestations in patients with skin complaints, and pet feeding.

There are two types of clinical demodicosis: Localized and generalized demodicosis. Ivermectin administration by mouth, injection, or absorption is an effective treatment [3,4]. Normally, this drug is rarely toxic, but retinopathy associated with ivermectin toxicosis in dogs has been reported [5]. A high dose is toxic in dogs carrying multidrug resistance mutation-1 gene mutations as well as the Collie breed [6].

In Thailand, the common drug for the treatment of demodicosis is ivermectin. Ivermectin is used to treat several parasites, such as helminths and arthropods. Ivermectin resistance has been reported for *Dirofilaria immitis* [7], *Rhipicephalus sanguineus* sensu stricto [8], and human *Sarcoptes scabiei* [9]. The most resistant are Acari mites. *Demodex* may be resistant to ivermectin. Moreover, in the case of secondary bacterial infection, dogs can develop pyoderma. The treatment comprises systemic antibiotics or topical antimicrobial therapy, such as benzoyl peroxide shampoo (2-3%), chlorhexidine shampoo (3-4%) [10], or ivermectin administered orally [11]. Co-administration with ketoconazole should be avoided because it could enhance the concentration of ivermectin [12].

Alternative treatments with natural compounds were shown to exhibit antimicrobial effects, efficacy, and improvement in the clinical signs of *Demodex*. There are various routes of administration, such as topical and oral. Natural plants and herbs have been reported, such as *Azadirachta indica* extract, Teeburb capsule (*Berberis aristata*, *Cedrus deodara*, *Curcuma longa*, and *Pueraria tuberosa*), and Demosymcan Gel [13-15].

Thus, this study was conducted to use natural compounds from *Dipterocarpus alatus*, *Rhinacanthus nasutus*, and *Garcinia mangostana. D. alatus* is a tropical forest tree that is commonly found in Southeast Asia. Dipterocarpol derivatives have anti-inflammatory, antiviral, and immunostimulation effects [16-18]. The oil from *D. alatus* (YN oil) was investigated. An individual tree can yield 0.5 L/day. The average price of YN oil on the local market is currently 228 Baht/kg ($7.00) with a range of 170-286 Baht/kg ($5.22–$8.78).

*R. nasutus* is a medicinal herb. Its common name is white crane flower (WC), and it is also found in Southeast Asia. WC can easily be propagated by stem cuttings. It has antioxidant and anti-inflammatory effects [19,20]. *G. mangostana*, also known as mangosteen (MG), is a tropical fruit found in Southeast Asia. It has antioxidant, antiparasitic, and anticancer effects [21-23].

This study aimed to determine the efficiency of YN oil, WC, and MG against canine demodicosis using a possible topical treatment.

## Materials and Methods

### Ethical approval and Informed consent

The study was approved by the Khon Kaen University Animal Ethics Committee (ACUC-KKU-19/2559). A consent form was signed by the dogs’ owners before the examinations. In brief, the dog’s history was taken, and a general physical examination was done. Dogs presenting with *Demodex* lesions were recorded. Dogs were fed normal food from the dogs’ owners and one boiled egg per day for 30 days. The boiled egg was used to control food intake from the diet. All dogs were bathed with WC shampoo (Neocare shampoo^®^, Vechmart, Thailand) every week for the prevention and control of *Malassezia pachydermatis* by the research team according to the manufacturer’s instructions. In the case of a cure rate <50%, the dog was treated with ivermectin after the end of the experiment within 2 months.

### Study period and location

The study was conducted from July to November 2018. The study was conducted in a village of Sakon Nakhon Province, Northeast Thailand (17°22′25.2″N, 103°43′12.9″E).

### Canine demodicosis

**Figure-1:**
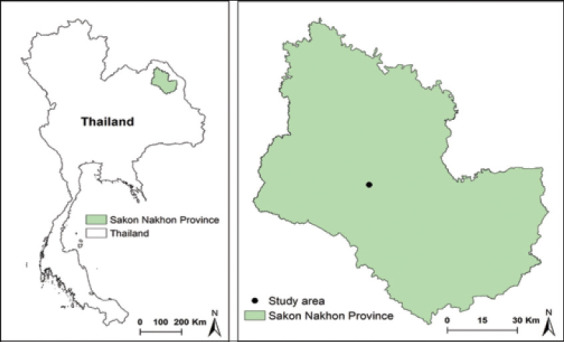
The location of the village in Phang Khon District, Sakon Nakhon Province, Northeast Thailand [Source: The administrative boundaries of Sakon Nakon Province and Thailand were downloaded from the DIVA-GIS database (http://www.diva-gis.org). A geographical information system (GIS) software ArcGIS 10.8 (ESRI, Redlands CA) was used to create a study map].

Twenty-five dogs (males and females) with demodicosis, aged 5-120 months, and with body weight of 7-20 kg, were used in the experiment, as shown in Figure-1. Dogs with skin lesions were confirmed to have demodicosis by deep skin scraping. The criteria for localized demodicosis were the following: (i) No more than four lesions with a diameter of up to 2.5 cm [24] and (ii) a range of four lesions to 50% coverage of the body surface with lesions [3].

**Figure-2:**
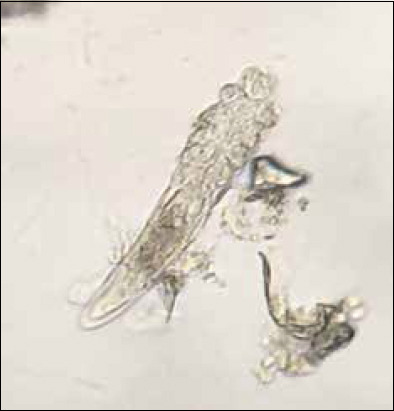
*Demodex* mites diagnosed with deep skin scraping.

The inclusion criteria for the canine demodicosis participants were as follows: (i) Age of more than 8 weeks, (ii) the presence of Demodex mites in skin scraping (Figure-2), (iii) clinically healthy except for clinical signs associated with localized demodicosis, (iv) no pregnancy history, and (v) no glucocorticoid treatment or any acaricide for at least 12 weeks before day 0. Dogs whose data could not be recorded until the end of the experiment were excluded from the study [25].

### Plant materials and preparation

YN oil was provided by Dr. Somporn Katekaew from the Faculty of Sciences, Khon Kaen University. The oil was collected from the stem of the tree. Briefly, the trees were drilled with holes, and then a container was used to collect oil from the holes. Chemical compositions were characterized by Asst. Prof. Dr. Ploenthip Puthongking from the Faculty of Pharmaceutical Sciences, Khon Kaen University [26]. The oil was melted in a beaker on a hotplate for 10 min at 60°C. Then, 60% YN oil in distilled water was prepared using a stand mixer for at least 30 min at 25°C.

WC was purchased from a local market in Khon Kaen Province and then extracted using a modified methanolic extract method [27]. Briefly, 500 g of fresh leaves were macerated with 1000 mL of ethanol at room temperature for 7 days. The macerate was filtered through 0.45 mm filter paper and evaporated using a rotary evaporator to remove the solvent. Then, 1.36% w/v WC was dissolved in distilled water and mixed well for at least 30 min at 25°C.

MG was purchased from a local market. MG extracts were prepared as in our previous study [22]. Briefly, fresh MG pericarps were incubated at 60°C, and then, the dried MG pericarps were macerated with ethanol at room temperature for 3 weeks. The macerate was filtered and evaporated. Subsequently, 10% w/v MG was dissolved in 4% dimethyl sulfoxide and mixed well for at least 30 min at 25°C. All preparations were kept in a refrigerator at 4°C. The YN oil, YN oil+WC, YN oil+MG, and YN oil+WC+MG had a pH of 5.

### Determination of components

YN oil was tested with a gas chromatograph-mass spectrometer [26]. The major components of the YN oil are sesquiterpenes, including α-gurjunene (30.31%), (−)-isoledene (13.69%), alloaromadendrene (3.28%), β-caryophyllene (3.14%), γ-gurjunene (3.14%), and spathulenol (1.11%). The presence of components was confirmed using thin-layer chromatography. This method was used for fingerprint analysis of the WC and MG extracts according to the previous studies before using them [23,28].

### Antioxidant activity determination

We confirmed the antioxidant activity of YN oil, MG, and WC. Free-radical scavenging activity was tested by 2,2-diphenyl-1-picrylhydrazyl (DPPH; Sigma-Aldrich, USA). The DPPH assay was modified from Aksoy *et al*. [29]. Briefly, the test samples were 100 μL of extracts (YN oil, MG, or WC) or a standard antioxidant compound (±)-6-hydroxy-2,5,7,8-tetramethylchromane-2-carboxylic acid (Trolox; Sigma-Aldrich, USA). The samples had various concentrations in 100 μL of the ethanolic DPPH• solution. Furthermore, 100 μL of ethanol and 100 μL of ethanolic DPPH• solution were used as a control. The solutions were incubated for 30 min at room temperature. The absorbance values were determined using a microplate reader (Sunrise™, Austria). The absorbances were measured at 540 nm. The antiradical activity was calculated as the inhibition percentage (I%) using the following equation:

(I%)=(absorbance control − absorbance sample)/absorbance control×100

We also calculated the half-maximal inhibitory concentration (IC_50_) value, which is the concentration of the extracts that caused 50% inhibition. The I% was plotted for five concentrations of extracts to obtain inhibition curves. A regression equation was estimated from the absorbance data points as follows:

I=mc+k

Where I is the I%; c indicates the concentration of YN oil, MG, or WC extract or Trolox; m is the slope; and k is the intercept. The IC_50_ values of Trolox, WC, and MG extracts were estimated to be 14.13, 174.27, and 84.38 μg/mL, respectively. However, the IC_50_ of YN oil suggested no antioxidant activity. The best result among the herbal treatments was observed for the MG extracts.

### Efficacy of *D. alatus* oil on canine demodicosis in vivo

A total of 25 dogs were divided into five treatment groups (five dogs/group): (i) A positive control (PC) group that received 10 mg/mL of ivermectin (Ivome^c®^, Merial Saude Animal Ltda, Brazil), (ii) dogs treated with topical YN oil, (iii) dogs treated with topical YN oil+WC, (iv) dogs treated with topical YN oil+MG, and (v) dogs treated with topical YN oil+WC+MG (Table-1). All dogs were shaved in the lesion areas. The fingertip unit of each treatment was applied to the skin lesions 3 times/week for 1 month. The dogs were photographed every day for 2 months. Pre-treatment photographs were taken at baseline, and post-treatment photographs were taken at the end of treatment for comparison. A skin scraping was not performed at the end of treatment.

**Table-1:** Treatment application and concentration for each experimental group.

Groups	Concentration	Application
i PC (ivermectin)	400 mg/kg	Subcutaneous injection, once a month
ii YN oil	60% YN oil in DW	Topical treatment, 3 times a week
iii YN oil+WC	60% YN oil and 1.36% w/v WC in DW	Topical treatment, 3 times a week
iv YN oil+MG	60% YN oil with 10% w/v MG in 4% DMSO	Topical treatment, 3 times a week
v YN oil+WC+MG	60% YN oil, 1.36% w/v WC, and 10% w/v MG in 4% DMSO	Topical treatment, 3 times a week

YN oil=*Dipterocarpus alatus* oil, WC=*Rhinacanthus nasutus* leaf, MG=*Garcinia mangostana* pericarps, PC=Positive control

### Clinical evaluation

The specific clinical parameters evaluated were alopecia, hyperpigmentation, and lichenification. The demodectic lesions on each dog were recorded, as shown in Table-2. In this table, the overall resolution of lesions is presented. Note that not all demodectic lesions were found on each dog.

**Table-2:** Percentage of dermatological lesions on dogs.

Dermatological lesions	Number of dermatological lesion dogs/Total number of dogs (%)
Alopecia	25/25 (100)
Hyperpigmentation	20/25 (80)
Lichenification	22/25 (88)

Alopecia was assessed as follows: 0, no alopecia (hair regrowth); 1, alopecia areas with partial hair regrowth; and 2, alopecia areas with no hair regrowth (Table-3) [30]. Hyperpigmentation and lichenification were assessed as follows: 0, no lesions (absent), and 1, lesion observed (Table-4). The efficacy evaluation was based on the percentage of reduction in dermatological changes on a dog after treatment. The numbers of dogs in a group affected by alopecia, hyperpigmentation, and lichenification were compared between pre-treatment and post-treatment.

**Table-3:** Clinical score of alopecia in all dog groups.

Clinical sign	Groups	Score	Number of dogs per score/Number of dogs examined					

			Day 0	Day 7	Day 14	Day 21	Day 28	Day 56
Alopecia	i PC	0	0/5	0/5	0/5	2/5	2/5	4/5
		1	0/5	2/5	4/5	3/5	3/5	1/5
		2	5/5	3/5	1/5	0/5	0/5	0/5
	ii YN oil	0	0/5	0/5	1/5	1/5	4/5	5/5
		1	0/5	4/5	3/5	4/5	1/5	0/5
		2	5/5	1/5	1/5	0/5	0/5	0/5
	iii YN oil+WC	0	0/5	0/5	1/5	2/5	4/5	5/5
		1	0/5	4/5	4/5	3/5	1/5	0/5
		2	5/5	1/5	0/5	0/5	0/5	0/5
	iv YN oil+MG	0	0/5	0/5	0/5	1/5	3/5	4/5
		1	0/5	3/5	4/5	3/5	2/5	1/5
		2	5/5	2/5	1/5	1/5	0/5	0/5
	v YN oil+WC+MG	0	0/5	0/5	0/5	0/5	4/5	4/5
		1	0/5	4/5	4/5	4/5	1/5	1/5
		2	5/5	1/5	1/5	1/5	0/5	0/5

0=No alopecia (hair regrowth), 1=Alopecia areas with partial hair regrowth, 2=Alopecia areas with no hair re-growth.

YN oil=*Dipterocarpus alatus* oil, WC=*Rhinacanthus nasutus* leaf, MG=*Garcinia mangostana* pericarps, PC=Positive control

**Table-4:** Clinical score of hyperpigmentation and lichenification in all dog groups.

Clinical sign	Groups	Score	Number of dogs per score/Number of dogs examined					

			Day 0	Day 7	Day 14	Day 21	Day 28	Day 56
Hyperpigmentation	i PC	0	0/4	0/4	0/4	0/4	1/4	3/4
		1	4/4	4/4	4/4	4/4	3/4	1/4
	ii YN oil	0	0/4	0/4	0/4	0/4	2/4	4/4
		1	4/4	4/4	4/4	4/4	2/4	0/4
	iii YN oil+WC	0	0/4	0/4	0/4	2/4	4/4	4/4
		1	4/4	4/4	4/4	2/4	0/4	0/4
	iv YN oil+MG	0	0/4	0/4	0/4	0/4	1/4	2/4
		1	4/4	4/4	4/4	4/4	3/4	2/4
	v YN oil+WC+MG	0	0/4	0/4	0/4	0/4	3/4	3/4
		1	4/4	4/4	4/4	4/4	1/4	1/4
Lichenification	i PC	0	0/4	0/4	0/4	0/4	1/4	3/4
		1	4/4	4/4	4/4	4/4	3/4	1/4
	ii YN oil	0	0/3	0/3	0/3	0/3	2/3	3/3
		1	3/3	3/3	3/3	3/3	1/3	0/3
	iii YN oil+WC	0	0/5	0/5	0/5	2/5	5/5	5/5
		1	5/5	5/5	5/5	3/5	0/5	0/5
	iv YN oil+MG	0	0/5	0/5	0/5	1/5	2/5	3/5
		1	5/5	5/5	5/5	4/5	3/5	2/5
	v YN oil+WC+MG	0	0/5	0/5	0/5	1/5	5/5	5/5
		1	5/5	5/5	5/5	4/5	0/5	0/5

0=No lesions (absent); 1=Lesion observed. YN oil=*Dipterocarpus alatus* oil, WC=*Rhinacanthus nasutus* leaf, MG=*Garcinia mangostana* pericarps, PC=Positive control

### Health observations

To evaluate the overall health, other clinical signs during treatment were recorded for all dogs, including allergy, lack of appetite, and lethargy.

### Statistical analysis

Cochran’s Q test was used to compare the dermatological changes of different time points for each treatment. A value of p<0.05 was considered statistically significant.

## Results

### General appearance

In Table-2, alopecia, hyperpigmentation, and lichenification were observed in 25 (100%), 20 (80%), and 22 (88%) dogs, respectively.

### Effect of *D. alatus* oil on clinical signs

**Figure-3:**
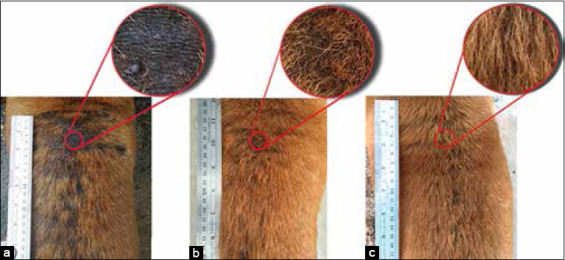
Occurrence of lesions in pre- and post-treatment of the positive control group. Back part; pre-treatment (a), 28 days post-treatment (b), and 74 days post-treatment (c).

**Figure-4:**
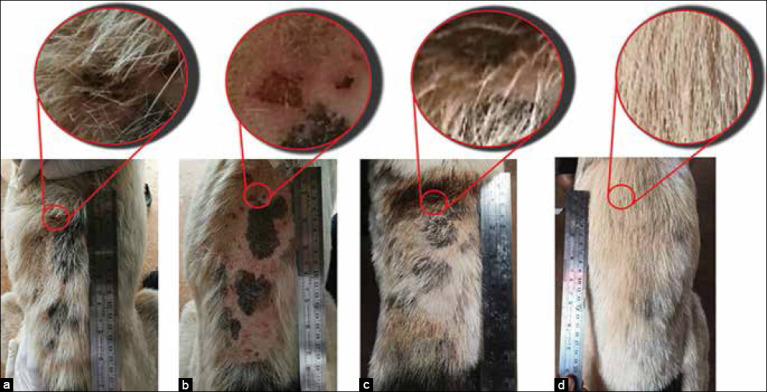
Occurrence of lesions in pre- and post-treatment of the YN oil group. Back part; pre-treatment (a), after shaving (b), 25 days post-treatment (c), and 56 days post-treatment (d).

**Figure-5:**
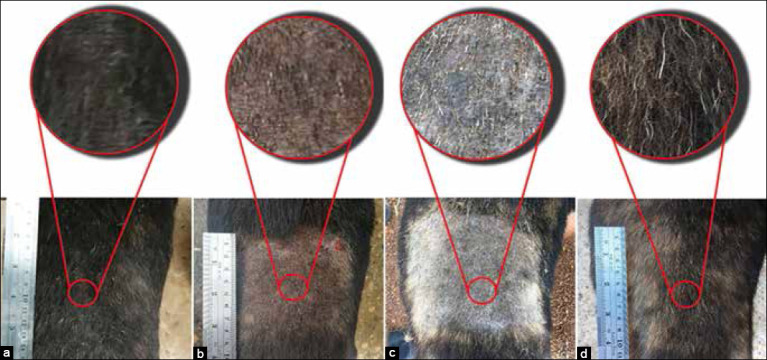
Occurrence of lesions in pre- and post-treatment of YN oil+WC group. Back part; pre-treatment (a), after shaving (b), 25 days post-treatment (c), and 56 days post-treatment (d).

**Figure-6:**
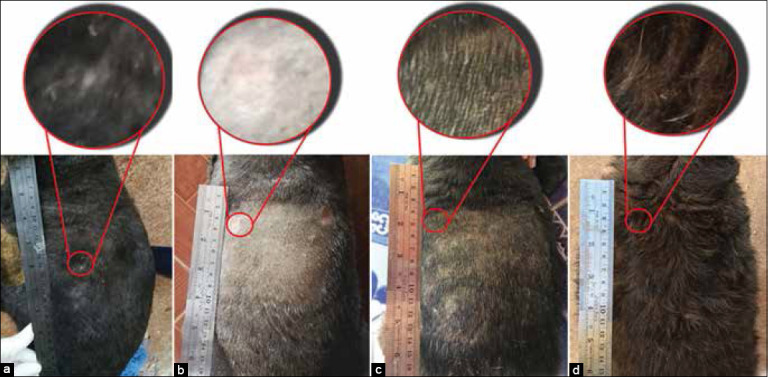
Occurrence of lesions in pre- and post-treatment of YN oil+MG group. Back part; pre-treatment (a), after shaving (b), 28 days post-treatment (c), and 56 days post-treatment (d).

**Figure-7:**
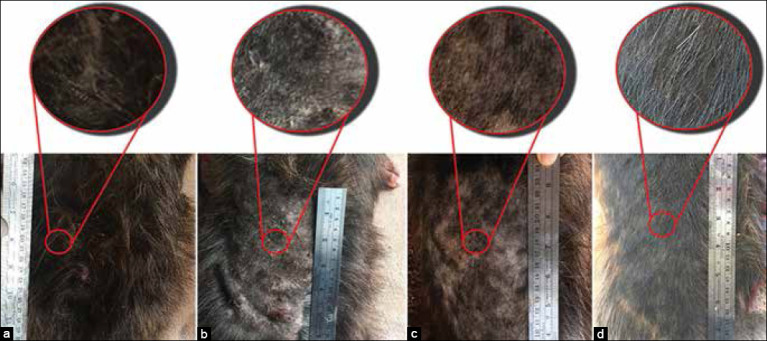
Occurrence of lesions in pre- and post-treatment of YN oil+WC+MG group. Back part; pre-treatment (a), after shaving (b), 28 days post-treatment (c), and 56 days post-treatment (d).

Clinical improvement was evident in dogs treated with YN oil+WC, including hair regrowth and reduced signs of hyperpigmentation and lichenification. The dermatological lesions were gradually reduced, and normal hair regrowth was observed at 14 days post-treatment with YN oil and YN oil+WC. On day 56, alopecia in all treatment groups significantly decreased (p<0.01). The PC treatment seemed to be effective, but 20% of the PC group presented with alopecia. By contrast, alopecia completely disappeared in dogs treated with YN oil and YN oil+WC (Table-5 and Figures-3-7).

**Table-5:** Percentage of reduction of dermatologic changes on the dogs after treatment (number of dogs/number of dogs per group).

Clinical sign	Groups	Day 0	Day 7	Day 14	Day 21	Day 28	Day 56	p-value
Alopecia	i PC	100% (5/5)	100% (5/5)	100% (5/5)	100% (5/5)	60% (3/5)	20% (1/5)	<0.01
	ii YN oil	100% (5/5)	100% (5/5)	80% (4/5)	80% (4/5)	20% (1/5)	0% (0/5)	<0.01
	iii YN oil+WC	100% (5/5)	100% (5/5)	80% (4/5)	60% (3/5)	20% (1/5)	0% (0/5)	<0.01
	iv YN oil+MG	100% (5/5)	100% (5/5)	100% (5/5)	80% (4/5)	40% (2/5)	20% (1/5)	<0.01
	v YN oil+WC+MG	100% (5/5)	100% (5/5)	100% (5/5)	100% (5/5)	20% (1/5)	20% (1/5)	<0.01
Hyperpigmentation	i PC	100% (4/4)	100% (4/4)	100% (4/4)	100% (4/4)	75% (3/4)	25% (1/4)	<0.05
	ii YN oil	100% (4/4)	100% (4/4)	100% (4/4)	100% (4/4)	50% (2/4)	0% (0/4)	<0.01
	iii YN oil+WC	100% (4/4)	100% (4/4)	100% (4/4)	50% (2/4)	0% (0/4)	-	<0.01
	iv YN oil+MG	100% (4/4)	100% (4/4)	100% (4/4)	100% (4/4)	75% (3/4)	50% (2/4)	>0.05
	v YN oil+WC+MG	100% (4/4)	100% (4/4)	100% (4/4)	100% (4/4)	25% (1/4)	25% (1/4)	<0.01
Lichenification	i PC	100% (4/4)	100% (4/4)	100% (4/4)	100% (4/4)	75% (3/4)	25% (1/4)	<0.05
	ii YN oil	100% (3/3)	100% (3/3)	100% (3/3)	100% (3/3)	33% (1/3)	0% (0/3)	<0.05
	iii YN oil+WC	100% (5/5)	100% (5/5)	100% (5/5)	60% (3/5)	0% (0/5)	-	<0.001
	iv YN oil+MG	100% (5/5)	100% (5/5)	100% (5/5)	80% (4/5)	60% (3/5)	40% (2/5)	>0.05
	v YN oil+WC+MG	100% (5/5)	100% (5/5)	100% (5/5)	80% (4/5)	0% (0/5)	-	<0.001

Group i (PC)=Dogs treated with ivermectin; Group ii (YN oil)=Dogs treated with topical YN oil; Group iii (YN oil+WC)=Dogs treated with topical YN oil+WC; Group iv (YN oil+MG)=Dogs treated with topical YN oil+MG; Group v (YN oil+WC+MG)=Dogs treated with topical YN oil+WC+MG. P*<*0.05 was considered significant.

YN oil=*Dipterocarpus alatus* oil, WC=*Rhinacanthus nasutus* leaf, MG=*Garcinia mangostana* pericarps, PC=Positive control

Hyperpigmentation was reduced by 50% after 21 days post-treatment with YN oil+WC. On day 28, dogs in the YN oil+WC group presented with 0% hyperpigmentation (p<0.01), whereas the treated dogs in the YN oil group showed 0% hyperpigmentation on day 56 (p<0.01). Hyperpigmentation was present in 25% of dogs in the PC and YN oil+WC+MG groups (p<0.05 and p<0.01, respectively). No statistically significant difference (p>0.05) was observed in dogs treated with YN oil+MG.

Lichenification lesions of dogs treated with YN oil+WC+MG and YN oil+WC were reduced by 20% and 40% after 21 days post-treatment, respectively. The YN oil+WC+MG and YN oil+WC groups showed a 100% total reduction in lichenification on day 28 (p<0.001). On day 56, dogs treated with YN oil and the PC group showed 0% and 25% reductions, respectively (p<0.05). No statistically significant difference (p>0.05) was observed in the YN oil+MG group.

### Health observations

After 2 months of observation, no allergy or clinical signs were observed on the skin, in behavior, or on the body.

## Discussion

YN oil, WC, and MG are very popular alternative herbal medicines as treatments for antibacterial effects, antifungal effects, liver diseases, and rheumatism. To the best of our knowledge, this study is the first to show that YN oil+WC is useful for localized canine demodicosis in dogs, with a focus on hair regrowth and the disappearance of hyperpigmentation and lichenification. The dermatological lesions were not detectable in dogs on day 56 post-treatment. Moreover, dogs treated with YN oil, YN oil+MG, and YN oil+WC+MG showed reduced dermatological lesions. No allergic or clinical signs were observed during treatment. The treatments were used 3 times/week for 1 month. Together with a WC, the shampoo was used for fungal prevention once per week.

Ivermectin is a common first choice for demodicosis treatment [31]. This drug acts as agamma-aminobutyric acid receptor agonist in the nerve cells of *Demodex*, resulting in parasite paralysis and death [32]. In the present study, the efficacy of the single dose of ivermectin injection was less than that of the alternative herbal medication of localized demodicosis treatment as evidenced by the dermatological lesions post-treatment (Table-5). A decrease in the overall dermatological lesions was noted, but there were still 20% alopecia, 25% hyperpigmentation, and 25% lichenification on day 56 post-treatment. By contrast, our present research shows success in 100% of three dermatological lesions curative with YN+WC treatment in dogs on day 56 post-treatment.

A previous study has shown that there are some herbal medicines for canine demodicosis. Herbal medicines are useful in the treatment of canine demodicosis using oral Teeburb capsules and the local application of skin healing spray [15]. *Withania somnifera* extract can be used for the treatment of canine demodicosis through antioxidant activity [33]. Nevertheless, there has been no report of the three herbs in this study for the treatment of canine demodicosis. They are not known to have miticidal properties, although there have been reports on their various properties. The clinical signs (erythema, alopecia, squamae, and hyperseborrhea) disappeared at 9 weeks after treatment with honey, propolis, apple vinegar, and extracts plant [13]. In the study results of Chakraborty and Pradhan [15], dogs were treated with Teeburb capsules twice daily orally for 30 days and skin heal spray twice daily for 20-30 days. Consequently, 50% of dogs showed negative for mite infection on the 45^th^ day. In this study, the dogs treated with YN oil+WC showed high efficacy of clinical signs and were cured within 8 weeks.

The major component of YN oil is alpha-Gurjunene. Alpha-Gurjunene has been previously reported to exhibit high antibacterial, anticancer [34], and tick-larvicidal effects [35]. WC contains rhinacanthin, which has various activities, such as anti-inflammatory [36], melanogenesis suppression [37], and antifungal activities. Finally, the major compound of MG is alpha-mangostin, which exhibits anti-inflammatory and antioxidant activities [22].

The evidence of *D. canis* infestation causing oxidative stress in dogs includes total antioxidant activity and superoxide dismutase [38]. We set out to determine the antioxidant properties of the three herbal medicines. The DPPH results of YN oil indicate no antioxidant activity, which is similar to a previous report [26]. For the other two herbs, the analysis of IC_50_ showed that *R. nasutus* and *G. mangostana* pericarp extracts have a high antioxidant activity, which is supported by previous studies [22,39]. This could be responsible for the added benefit of adding these two herbs in this study.

A previous *in vitro* antifungal study showed that 13.6 mg/mL of WC extract exhibited inhibition of *Trichophyton mentagrophytes* and *Microsporum gypseum* until 3 days, but 27.2 mg/mL of WC showed fungicidal activity, which is similar to the previous report [40]. Thus, we prepared WC shampoo to prevent and control fungal infection during the experiment. In the present study, the dogs treated with YN oil, YN oil+WC, YN oil+MG, and YN oil+WC+MG showed hair regrowth on day 56 post-treatment, which may result from the inhibition of the proliferation of the mites. The pathogenic mechanisms of alopecia in canine demodicosis are cutaneous barrier rupture by the proliferation of the mites [41]. A previously reported essential oil from *Drimys brasiliensis* Miers has α-gurjunene compounds. They exhibited larvicidal activity against cattle ticks and brown dog tick [35]. Ticks are a type of Acari mite. Alpha-gurjunene compounds were found in YN oil. Hence, the mechanism of action of this compound may act on *Demodex* mites when considering that all dog groups treated with YN oil had regrown normal skin.

As previously reported, hyperpigmentation from canine demodicosis is caused by increasing melanocyte activity in the epidermis [42]. Our results showed that for the dogs treated with YN oil, hyperpigmentation disappeared on day 56 post-treatment. This finding is supported by a previous report that showed that sesquiterpene lactones inhibited melanin production [43]. In addition, another study found that WC extracts reduced melanogenesis [37]. This is in agreement with our results since hyperpigmentation disappeared on day 28 post-treatment in the dogs treated with YN oil+WC. When compared with dogs treated with only YN oil, the dogs’ skin improved more with YN oil+WC than with only YN oil. This suggests that a combination of the YN oil and WC could highly reduce melanogenesis.

Our results showed that YN oil, YN oil+WC, and YN oil+WC+MG significantly decreased lichenification. Lichenification is the thickening and hardening of the skin from chronic disease. This result is in agreement with a previous report that salicylic acid solutions could produce histologic changes. The skin became thinner [44]. This result may be a consequence of the acidity of the herbal medication. All of the herb treatments were acidic with pH 5. This pH may affect the epidermis of demodicosis skin, which became thinner after treatment. Furthermore, YN oil can increase skin permeability [45].

Another factor of canine demodicosis from mite infestation is nutritional conditions. Dogs with poor body conditions show higher mite infestation when compared with dogs with normal body conditions [46]. In this study, all dogs showed signs of malnutrition. Thus, we fed each dog one boiled egg every day until 30 days to boost their nutritional condition. Eggs are a high source of protein, riboflavin, and selenium, and boiled eggs are a dietary supplement for dogs.

## Conclusion

In conclusion, the dermatological lesion (alopecia, hyperpigmentation, and lichenification) disappeared within 56 days in a dog treated with YN oil+WC. Our present data demonstrate that YN oil+WC can be used as an alternative treatment for localized canine demodicosis. The benefits of these herbal medications are that they are cheap and easy to find, have simple preparation, and could reduce the medication cost for treatment. No visible adverse effects were noticed from these herbal medications.

## Authors’ Contributions

AA, PB, BP, PS, RA, SB, OP, and CE: Data curation, investigation, methodology, and formal analysis. AA and TB: Conceptualization and project administration. TB and WZ: Supervision and visualization. AA, PR, AS, PL, and TB: Drafted and revised the manuscript. All authors read and approved the final manuscript.
